# Utility of the US Metabolic Syndrome Severity Calculator for Group-Level Comparison in Estonia

**DOI:** 10.3390/medicina62020363

**Published:** 2026-02-12

**Authors:** Ülle Parm, Anna-Liisa Tamm, Heete Kuuskla

**Affiliations:** Physiotherapy and Environmental Health Department, Tartu Applied Health Sciences University (TAHSU), 50411 Tartu, Estonia; ulle.parm@tartuh.ee (Ü.P.);

**Keywords:** metabolic syndrome, metabolic syndrome severity score, calculator

## Abstract

*Background and Objectives*: Metabolic syndrome (MetS) is globally prevalent, highlighting the need for easy risk identification. This study determined the MetS severity score in different groups of women in Estonia using a calculator developed based on the US population. *Materials and Methods*: The sample included 153 women (20–50 years): commercial diet plan (CDP) users, physically active women (physical activity, PA, >5 h/week), and a control group (CG). The factors assessed included age, gender, body mass index (BMI), systolic blood pressure, high-density lipoprotein (HDL) cholesterol, triglycerides, and fasting glucose, yielding a MetS percentile and Z-score. Statistical analyses comprised descriptive statistics, *t*-tests, and χ^2^ tests. Differences were considered statistically significant at *p* ≤ 0.05 and at <0.017 when comparing the three study groups. *Results*: The PA group was younger and had a lower BMI (*p* < 0.05), while the CDP group had lower HDL cholesterol levels (*p* < 0.017). MetS percentiles were higher in the CDP group (vs. PA *p* = 0.002; vs. CG *p* = 0.016). Despite having a lower Z-score than US women, the CDP group showed higher MetS severity levels (>1). *Conclusions*: The US calculator is also suitable for comparing different MetS risk study groups located outside the US, but for assessing individual MetS risk, it must be adapted and validated for the relevant population.

## 1. Introduction

Metabolic syndrome (MetS) is a condition characterized by a constellation of metabolic abnormalities, including insulin resistance, central obesity, hypertension, and dyslipidemia, and it poses a significant risk for the development of atherosclerotic cardiovascular diseases and type II diabetes mellitus. It has also been shown that the incidence of cerebrovascular (stroke) or peripheral vascular diseases [1], fatty liver disease [2], hepatocellular carcinoma, pancreatic cancer, and systemic inflammation [3] is increased in individuals with MetS compared to the general population.

In Europe and the US, around one-fourth of the general population is reported to have MetS [1]. Data vary, with Liang et al. [4] reporting a prevalence of 41.8% in the US in 2017–2018. However, the use of different definitions causes variations in results. For example, Finns [5] compared the distribution of subjects according to the definitions of the World Health Organization (1998), the National Cholesterol Education Program Adult Treatment Panel III (2004), the International Diabetes Federation (2005), and the Joint Interim Statement (2009). They found that the prevalence of MetS varied significantly between these four definitions, at 17.7%, 33.3%, 41.5%, and 43.0%, respectively. In addition to diagnostic criteria, the prevalence of MetS varies globally according to factors such as living in an urban or rural area and demographic characteristics such as age, gender, race, and ethnicity [3]. Nevertheless, it has increased dramatically in recent decades, likely due to the rise in the number of people with obesity, affecting more than one-fifth of Americans and Europeans [1].

The Adult Treatment Panel III guidelines state that a diagnosis of MetS should be made in women when any combination of three or more of the following five risk factors is observed: (1) a waist circumference (WC) ≥ 88 cm (35 inches), (2) a fasting blood glucose (GL) ≥ 100 mg/dL, (3) a serum triglyceride (TG) level ≥ 150 mg/dL, (4) HDL < 50 mg/dL, or (5) a systolic blood pressure (SBP) ≥ 130 mmHg [1,3,6]. However, according to more recent studies, population and country specifications should be considered when determining WC [1,3]. Additionally, it has been shown that different WC measurement sites yield different results when determining the prevalence and severity of MetS [7]. In the context of MetS, studies use different parameters or biomarkers of associated diseases such as high-sensitivity C-reactive protein, uric acid, fasting homeostatic model assessment of insulin resistance (HOMA-IR), apolipoprotein B (APOB), apolipoprotein A1 (APOA1), glycated hemoglobin HbA1c [6,8], the low- and high-density lipoprotein cholesterol ratio (LDL/HDL), and the APOB/APOA1 ratio [9], as well as parameters such as gender, race, or ethnicity [10,11,12].

Obesity and insulin resistance play important roles in the etiology of MetS [13]. The pathogenesis of MetS has not been fully characterized, but it may be influenced by various genetic and environmental factors, including overeating, smoking, stress, and inactivity [1,3]. Therefore, MetS and related diseases can be largely prevented through lifestyle changes, such as weight loss, dietary modification, and physical activity (PA) [3], which is why it is important to identify MetS risk as early as possible. MetS risk assessment can be used as a tool for screening people at increased risk and, if necessary, directing appropriate treatment [12,13].

Recent efforts have focused on developing MetS severity scores [14] that can be used to predict the risk of cardiovascular disease or type 2 diabetes. Prospective population-based studies should be conducted to assess their accuracy and validity in predicting disease [15]. The scores should take into account weight, sex, age, and ethnicity; suitable MetS components should be identified; and methods should be developed based on a large dataset [6,12,16]. Calculators provide a rapid and simple means of assessing MetS severity; however, unfortunately, it does not have a universal definition. Some researchers have defined it as the sum of the z-scores of the MetS components [16], while some calculators are designed for narrow age ranges [17] or do not allow accurate data to be entered [18].

Based on a representative sample of the US population and these indicators, a calculator has been developed for the US population [2], and it provides two different outputs: MetS severity score (Z-score), which is a standardized value indicating how many standard deviations an individual’s MetS severity is above or below the population mean, and the percentile (MetS%), which reflects the proportion of the population with a MetS severity lower than the examined individual. The MetS severity score is calculated via reliable statistical processes (mainly confirmatory factor analysis) and validated equations. The calculation requires data on the five components of MetS, namely, SBP, TGs, HDL cholesterol, GL, and WC, although the latter can be replaced with body mass (BM) status.

As Estonia is a small country for which developing a separate calculator would be time-consuming and resource-intensive, it should first be assessed whether the version intended for use in the United States could also be used here. The aims of this study were to determine the MetS severity scores (Z-score and percentile) of groups of women with different expected risk levels (individuals who are physically active, those following a commercial diet plan (CDP), and a control group (CG)) using a calculator developed based on the US population; to examine the correlations of the indicators used in the calculator among women who participated in this study; and to compare the results between different study groups.

## 2. Materials and Methods

The data used in this study were collected as part of an applied study conducted at Tartu Applied Health Sciences University (TAHSU), which was approved by the Research Ethics Committee of the University of Tartu (Estonia; protocol no.: 340/T-2, 19 April 2021). This is a cross-sectional and descriptive study based on a volunteer sample, and it was conducted in June 2021 at TAHSU.

### 2.1. Participants

Information about participant recruitment for this study was disseminated through the university’s social media channels (including Facebook). The sample consisted of 161 Estonian-speaking women, aged 20–50 and living in Estonia (excluding menopausal women), who were divided into three study groups: (1) a group consisting of women who had been following CDP Fitlap [19] for at least the past three years; (2) a group consisting of physically active women whose weekly active exercise load was >5 h (or >3000 metabolic equivalent, MET-minutes/week); and (3) a CG consisting of women who had not followed any diet plan in the past three years. The CDP Fitlap has been offered since 2015, and, to date, >160,000 adult women in Estonia have followed it. Ninety percent of followers aim to lose weight, and, although the diet is based on national dietary recommendations, its carbohydrate content is lower (ca. 20%), and its protein content is higher (40%) [20]. The women in the PA group trained according to a specific training plan and under the guidance of a coach and competed at a national or international level.

**Figure 1:**
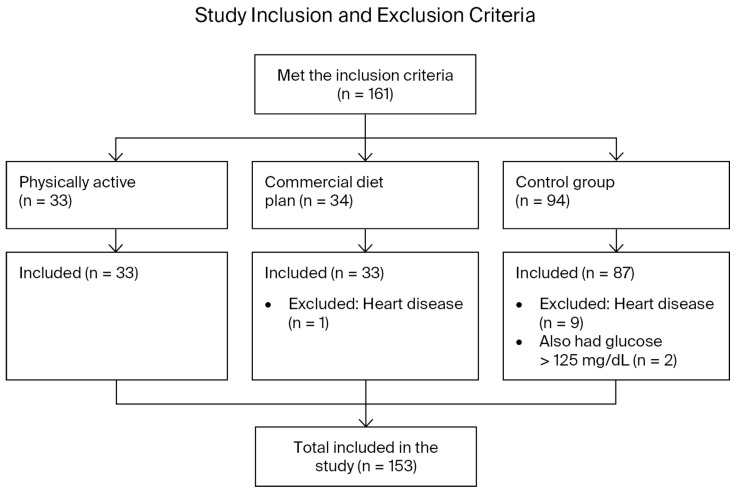
Study inclusion and exclusion criteria.

The final study group consisted of 153 participants (Figure 1). In accordance with the calculator requirements [2], data from ten women were excluded from the analysis due to the presence of heart disease. Among them, one was a member of the CDP group (with a positive Z-score), and nine were members of the CG (four with a positive Z-score), two of whom also had fasting blood sugar levels above 125 (to rule out latent diabetes). Notably, there were no pregnant women among the participants, and none were using antihyperlipidemic or antidiabetic medications.

### 2.2. Study Design

Individuals interested in participating in this study contacted the principal investigator by e-mail to arrange a meeting at TAHSU, where they were first given an overview of the study and signed a bilateral informed consent form. Body height and weight were measured by a trained specialist at the Institute of Sport Sciences and Physiotherapy of the University of Tartu according to the standard technique and with calibrated instruments. Height was measured to the nearest millimeter using a Harpender metal anthropometer, and body mass was measured to the nearest 0.1 kg using digital scales (A&D Instruments Ltd., Abingdon, UK). Blood pressure (BP; systolic and diastolic) was measured at TAHSU in the resting condition using a sphygmomanometer. The participant was asked to sit down and relax for about three minutes before the procedure. The upper arm was tested rested on a table, at about the same height as the heart, while the reading was carried out. BP was measured three times, and the mean value of the first and second measurements was used [5].

For laboratory tests, a registered nurse collected 14.5 mL of blood (2 × 5 mL of unadulterated + 2 mL of glucose inhibitor in a tube and 4.5 mL of EDTA blood) after overnight fasting. For blood collection, the subject was asked to sit comfortably in a chair; if necessary, they were sedated (if they wished, the activity was interrupted, and blood was not collected); and the hand was placed comfortably on an armrest. For clinical chemistry analyses, serum was separated via centrifugation and stored at −20 °C until analysis. Analyses were performed in the TAHSU laboratory using clinical chemistry analyzers: a BS 120 (fully automatic clinical chemistry analyzer), a Cobas c111 (fully automatic clinical chemistry analyzer; Roche Diagnostics International AG, Rotkreuz, Switzerland), and an IMMULITE 2000 (Siemens, Munich, Germany). For this study, TG, HDL cholesterol, and fasting GL levels were determined.

Energy and nutrient intakes were estimated as the average of three twenty-four-hour dietary records (including two weekdays and one weekend day). Participants were instructed to maintain their usual dietary habits before this study. The Nutridata System for Research (the Estonian Health Development Institute) [21] was used to analyze the data collected via dietary records. Only the dietary energy value data obtained were used in this study. PA levels were assessed using the International Physical Activity Questionnaire Short Form (IPAQ-SF, validated in Estonian) [22], which contains questions about 3 specific types (high, moderate, and low) of activity performed during the last 7 days for healthy adults. Moderate PA is defined as achieving a total of at least 600 MET-minutes per week. High PA is defined as achieving either a minimum of 1500 MET-minutes weekly through vigorous-intensity activities over at least three days or a total of at least 3000 MET-minutes through any combination of walking, moderate-intensity, or vigorous-intensity activities over seven days [23].

### 2.3. Calculating the Risk of Metabolic Syndrome

The US calculator [2] was used to calculate the severity of MetS. It provides two different outputs: the MetS severity score (Z-score) and the percentile (MetS%) based on the US population. The calculator is suitable for people aged 20–64 years. Subjects must not be pregnant, have diabetes or be suspected of having diabetes (fasting GL level > 125 mg/dL), have heart disease (congenital defects, ischemic disease, or previous infarction), or use hyperlipidemia or diabetes medications [10]. In Estonia, mmol/L is used to express triglyceride, glucose, and HDL cholesterol values, but mg/dL is required for the calculator; therefore, conversions were performed, where the glucose value was multiplied by 18, triglycerides by 88.57, and HDL cholesterol by 38.67.

To develop the US calculator [4], data were obtained from NHANES (1999–2010) [10], which was based on a large sample (*n* = 6870); thus, the calculator is widely accepted. MetS% indicates the proportion of the US population that has a lower percentage than a given individual. The calculator uses body mass index (BMI; BM and height or WC), SBP, TG, GL, and HDL cholesterol, as well as age, gender, and ethnicity and race: Hispanic, non-Hispanic black, and non-Hispanic white [2]. The latter were also examined in our research group comparison. The possibility of using BMI instead of WC allowed us to conduct this study, although the absence of WC measurement is a fundamental design limitation, as it is a key diagnostic criterion for MetS and closely associated with visceral adiposity [24]. Based on the above indicators, the calculator also provides a Z-score, which essentially enables a comparison with the distribution of the US population. All participants were white European women.

The MetS Z-score is not calculated directly like other Z-scores but is derived using confirmatory factor analysis, which considers the correlations between the same five MetS components mentioned above. Technically, it is the numerical value of the standard deviation. A Z-score of 0 indicates that the individual’s value is equal to the population mean, positive values indicate a higher risk of MetS, and negative values indicate a lower risk. For example, if Z = 2 (equivalent to the 97.7 percentile), then 97.7% of the population has a lower MetS severity than the given individual, and if Z = −2 (equivalent to the 2.28 percentile), then only about 2% of the population has a lower MetS value. However, the score only allows for a comparison with the US population, and, currently, the calculator does not provide detailed information about the risk of cardiovascular disease and diabetes [2].

### 2.4. Statistics

The programs MS Excel 2019 and Sigma Plot for Windows version 11.0 (Systat Software Inc., San Jose, CA, USA) were used for data processing. Descriptive statistics are used to describe the data characterizing all three groups. For comparison, ANOVA was first used for numerical values, the results of which were specified with a *t*-test or Mann–Whitney test; for non-numerical values, the χ^2^ or Fisher Exact test was used. When parameters were not normally distributed, medians are used for the sake of consistency in the table. To check the correlation of the parameters under study (as well as to select the necessary parameters of the calculator), the Spearman correlation coefficient was used. Differences were considered statistically significant at *p* ≤ 0.05. According to the Bonferroni method, an adjusted *p*-value of 0.017 was used to compare the three study groups.

## 3. Results

### 3.1. Baseline Data of Different Study Groups and for Calculating Metabolic Syndrome Severity

The presence of MetS is confirmed if three of the five factors (WC, SBP, TGs, GL, and HDL) do not meet the requirements. As WC measurements were not available, the calculator was used to determine MetS severity scores based on BMI values. When we considered a BMI value above 24.9 as an indicator of MetS instead of WC, at least three values did not meet the requirements in 11 subjects, 4 of whom were in the CDP group, and 7 were in the CG. This indicates the possible presence of MetS, and these study participants were treated as members of the corresponding study group and are not reported separately.

Table 1 shows the medians of the data required for calculating MetS severity using a calculator, as well as the PA and dietary energy data, as the corresponding numerical values were mostly not normally distributed. The representatives of the PA group were younger, and their BM, BMI, and PA level were higher than those of the representatives of the CDP group and CG. BMI was higher in the CDP group than in the PA group. Women in the CDP group had a lower mean HDL cholesterol value and a higher mean PA level than those in the CG. The other parameters did not differ between the groups.

### 3.2. Correlation Between Parameters Used When Using the Calculator

The correlations of the parameters required for calculating MetS severity are presented in Table 2. As expected, there was a negative correlation between HDL cholesterol (a factor unfavorable for MetS, although only in terms of BMI) and a positive correlation between the remaining parameters (favorable for MetS). However, not all parameters correlated with each other. For example, HDL cholesterol levels did not correlate with age, fasting glucose levels, or SBP, and triglyceride levels did not correlate with age or glucose levels.

### 3.3. Metabolic Syndrome Severity Percentile (Mets%) of Different Study Groups

**Figure 2:**
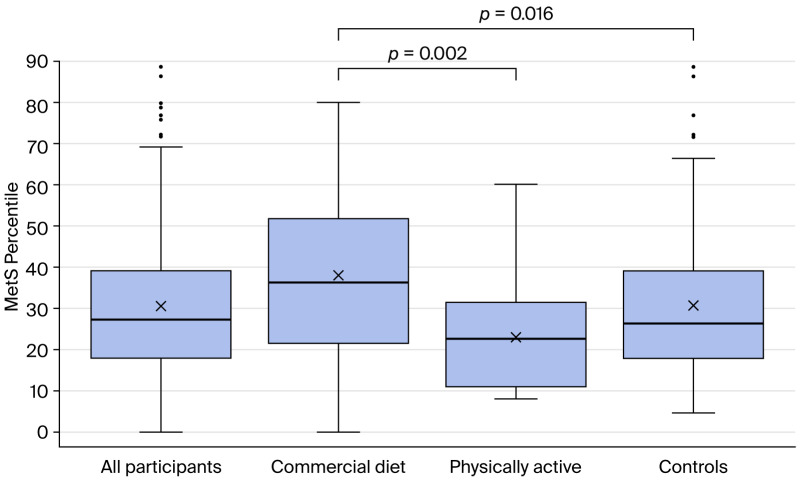
MetS percentile (Mets%) of different study groups (ANOVA/*t*-test; Bonferroni correction).

Figure 2 shows the distribution of MetS% among the study groups. The CDP group has a statistically higher MetS% than the PA group and CG, although the difference between the CDP group and the CG is only evident when comparing these two groups.

### 3.4. Comparison of Z-Scores Between Different Estonian Study Groups and with US Women

**Figure 3:**
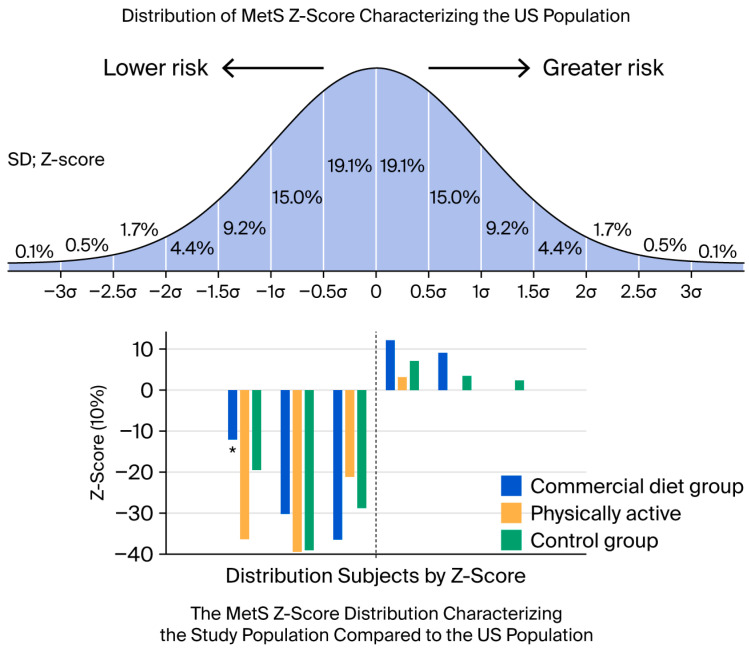
Distribution of different groups of Estonian women participating in this study according to Z-scores, considering those developed based on US population data. * *p* = 0.004 (χ^2^ test).

The distribution of the Z-scores of the study groups and their placement on the Z-score scale developed for the US population [2] are presented in Figure 3. The Z-score (essentially the standard deviation value) of the women participating in the study ranged from −1.66 to 1.21, and only 19 had a value > 0. Thus, the value of this indicator in the women in the PA group and CG was lower than the average of the entire US population. The same can be said visually for the CDP group. However, 7 out of the 33 women in this group had a Z-score > 0, and these values were more similar to those of the US population than to the other groups. If we only look at the distribution of the different study groups of Estonian women according to the Z-score developed for the US population, then it can be observed that, among those with a value < −0.1, there were more participants from the physically active group than from the CDP group.

## 4. Discussion

It has been previously shown that MetS and cardiovascular diseases are significantly associated and that determining MetS severity is important for implementing preventive and therapeutic measures [13,25]. It is also important for the early assessment of the risk of type 2 diabetes [13,26]. The MetS severity score is considered a sensitive method for identifying high-risk patients, and, thus, it can be used to motivate lifestyle changes and monitor treatment progress [27,28]. Therefore, we determined MetS severity by comparing physically active individuals, CDP followers, and a CG to assess the suitability of the US calculator, using Estonian women as an example.

The results of this study show, as expected, that the PA group had lower MetS risk scores, especially compared to the CDP group. It is believed that the pathogenesis of MetS involves several complex pathways; additionally, among the complex interactions with various genetic and environmental factors, PA plays an important role in influencing its development [3]. Previous studies [29] have shown that increasing PA improves metabolism and that early initiation of exercise leads to better physical fitness, glycemic control, and weight loss and improved lipid profiles. PA and an active lifestyle have a preventive effect on MetS and related diseases [1,12,30], and excessive sitting has been found to be a major risk factor for developing MetS in the future [3].

The MetS risk-related indicators of the CDP group were worse than those of the other groups. The commercial Fitlap diet plan is described in [19]; ninety percent of its followers aim to lose weight. Although this diet is based on national dietary recommendations, its carbohydrate content is lower (ca. 20%), and its protein content is higher (40%). This study group had a higher BM and BMI, and we also previously showed in an almost identical study group [20] that, although their dietary energy intake did not differ from that of the CG, their dietary intake of calcium and magnesium, BM, and bone mineral density (BMD) were higher. As the study subjects were still attempting to lose weight, this can be explained by the positive effect of a higher BMI on BMD [31], which has been shown even in older adults [32]. However, increased adipose tissue can lead to systemic inflammation and the development of MetS [3], an increased risk of which was also observed in this study group.

Abdominal obesity is most strongly associated with MetS and primarily manifests as an increase in WC [27]; it is also one of the criteria for MetS [1,3,6]. Unfortunately, we did not examine it in this study, as it was not required for the calculator used. However, a study conducted in West Asia showed that, in women, following an age of 40–60, WC was the second most important factor in determining MetS [16]. It has been proposed as a marker of visceral adiposity, but it may also vary with BMI. However, the correlation between WC and visceral adiposity depends on age and gender [3]. Additionally, a study [7] that assessed the association between WC measured at nine different sites and individual risk factors for MetS yielded mixed results, indicating the need for consensus regarding the measurement site. Therefore, we trust the results of the US calculator, which considered BMI values.

A strong positive correlation has been shown between MetS severity score and BMI, which, in turn, indicates an association between MetS and obesity [8,28]. Thus, our study results were expected to agree with those obtained in the US population. According to data from the Adult Population Health Behavior Survey conducted in the spring of 2024, 52% of Estonian people aged 16–64 were overweight or obese (BMI > 25) [33]. However, the 2017–2018 National Health and Nutrition Examination Survey (NHANES) found that this rate in the US was 74.6% [34]. Today, in America and other developed countries, a major cause of weight gain is neuroendocrine dysregulation [35]. In a weight loss diet, in addition to reducing calories, it is recommended to eat less saturated and trans fats, cholesterol, sodium, and simple sugars and to consume fruits, vegetables, and whole grains [27].

As MetS is associated with BMI, elevated BP, high triglyceride and blood sugar levels, and low HDL cholesterol levels [1,3,6,27], which are mostly interconnected, it is important to monitor these parameters and keep them within normal levels. To achieve this, lifestyle changes are recommended, including physical exercise, a healthy diet, quitting smoking, and getting enough sleep [1,8]. For example, in the US, it has been proposed that primary care physicians should screen all adults for obesity and recommend lifestyle changes, as approximately 95 million adults could benefit [35]. In particular, they should encourage the adoption of exemplary dietary and PA habits. Lifestyle modification programs typically prescribe, among other recommendations, 150–180 min per week of moderately vigorous aerobic activity, such as brisk walking or cycling [35]. Furthermore, each clinical component of MetS can be addressed with PA, which is a low-cost and therefore easily accessible method for prevention and treatment [29].

In the US, the most impressive evidence for the health benefits of moderate (5–10%) weight loss comes from lifestyle interventions designed to prevent progression from impaired glucose tolerance (i.e., prediabetes) to type 2 diabetes [35]. MetS is associated with insulin resistance, which can lead to increased blood glucose levels and weight gain [3]. The calculator cannot be used for individuals with diabetes or fasting blood sugar levels > 125 mg/dL, so we cannot conclude on the respective associations. However, two subjects were removed from this study because their glucose levels exceeded this value. Although obesity and insulin resistance are strongly associated with increased BP, it is believed that hypertension is less “metabolic” than other components of metabolic syndrome [27]. In our study, the median BP in each group remained below the elevated limit [36].

MetS risk should be examined even in young people, as high correlations between childhood and adulthood scores were previously found in the same individual, indicating the persistence of metabolic phenotypes. A link was also found between higher childhood MetS severity scores and the development of either cardiovascular disease or type 2 diabetes in adulthood [12]. A simpler method would be to use appropriate calculators. Assessment of MetS and MetS severity scores varies somewhat across studies and calculators. For example, for some calculators, it is necessary to enter the ranges of parameter values [18], or data on eating habits, sleep, PA, etc., must also be entered [37]. However, after entering numerical values, some provide qualitative results regarding the risk [38]. Some articles [26] consider the nationality (for example, West Asian) of the respondents and describe the specifics of calculating their scores [16].

The calculators used in our study [2] and others [6] involve the use of confirmatory factor analysis, which examines how the various MetS components (obesity, BP, TGs, HDL cholesterol, and fasting GL) correlate with one another. This also allows for the correlations between the MetS components to be assessed based on sex and race/ethnicity. The calculator used in our study allows for WC to be replaced with BMI, which is calculated from BM and height. Furthermore, it is freely available and reliable but is based on data from US residents. In our study, we initially limited ourselves to simply calculating correlations between corresponding parameters. Although the results showed an association between most of the parameters studied, HDL cholesterol levels did not correlate with age, fasting GL levels, or BP, and TG levels did not correlate with age or GL levels. Although the calculator uses classic MetS parameters, it is possible that other parameters are more suitable for Estonian individuals. However, in order to clarify and confirm this, the calculator needs to be validated on a population basis. Although Gurka et al. [10] found that C-reactive protein (CRP), HOMA-IR, and uric acid are not ideal for determining MetS scores, Honarvar et al. [16] considered the lack of data on markers such as CRP, uric acid and HbA1c as a drawback in assessing associations for their MetS score.

As expected, the risk of MetS was lower in the PA group and higher in the CDP group (in those who lost weight). It can therefore be concluded that the US calculator is also suitable for comparing MetS severity in study groups with different expected risk levels in other countries. MetS calculators are typically developed using data from a specific population or geographic region, and their accuracy may decrease when applied to other populations; therefore, sex-, ethnic-, or region-specific adaptation is often required prior to broader clinical implementation [39]. Both internal and external validations, as well as assessment of prognostic performance in relation to cardiometabolic outcomes, are critical components of calculator development [40]. Consequently, conclusions based on a calculator developed in the United States cannot be reliably applied at the individual level in other countries.

This study has several important limitations that should be explicitly acknowledged. First, waist circumference (WC) was not measured; WC is a core component of MetS definitions and the original MetS severity score, and substituting BMI for WC weakens the physiological interpretation of the results and may reduce the validity of the score in this cohort. Second, the cross-sectional design prevents an assessment of predictive validity for future cardiometabolic outcomes; the findings therefore pertain to group-level discrimination rather than individual risk prediction. Third, we did not perform latent construct testing (e.g., factor analysis) or recalibration to verify that the MetS construct behaves similarly in the Estonian population; such analyses (calibration and population-specific validation) are warranted in future work.

Fourth, the recruitment strategy and study sample may have introduced bias and limited generalizability: participants were self-selected via social media, randomization was not employed, and the PA and CDP groups were relatively small. Fifth, several relevant measures were not available or controlled for, including HbA1c and HOMA-IR, detailed quantification of CDP PA levels and supplement or dietary intake, and menstrual cycle phase—factors that can influence glucose and lipid markers. Inclusion of a structured comparison group combining objective physical activity and dietary measures would have strengthened the design. Finally, the exclusively Estonian sample limits extrapolation to other ethnic groups. It has been shown that MetS components can vary within the same population and influence the variability of MetS severity score equations in different ethnic groups [41]. Future studies should include larger, more ethnically diverse samples and cross-population comparisons, using appropriate statistical methods, to determine whether the observed associations and score performance can be generalized to other populations.

## 5. Conclusions

This study demonstrates the applicability of the US MetS severity calculator in a study sample. As expected, the calculator shows more favorable results for physically active women and less favorable results for women with a higher BMI. MetS severity was higher in those who are theoretically at greater risk. Therefore, it can be assumed that the US calculator is also suitable for comparing different MetS risk study groups located elsewhere. To assess individual MetS risk, the calculator should be adapted and validated for the Estonian population.

## Figures and Tables

**Table 1:** Medians of parameters required to calculate MetS severity in different study groups.

Parameter	Physically Active Group		CDP Group		Control Group		All Participants	
	Median	IQR	Median	IQR	Median	IQR	Median	IQR
*n*=	33		33		87		153	
Age (y)	29.0°●	22.0–39.0	37.0	31.5–43.3	38.0	27.0–47.0	37.0	26.0–45.0
Energy from diet (kcal)	1895.1●	1762.4–2297.3	1848.2	1627.3–2048.0	1754.8	1507.6–2035.2	1832.9	1592.3–2078.9
Physical activity (MET-min/week)	5508.0°●	3478.5–8252.6	3813.0●	1902–5181.8	2772.0	1632.8–5223.0	3439.5	1956.8–5905.5
Body mass (kg)	62.0°●	57.3–69.5	73.9	68.0–84.4	65.3°	59.0–75.9	66.9	59.8–76.4
Height (cm)	170.0	165.0–174.0	170.0	164.8–172.3	168.0	164.6–172.0	168.9	165.0–172.0
Body mass index (kg/m^2^)	21.4°●	20.3–24.2	26.4	23.7–28.3	23.2	20.9–27.2	24.4	21.0–27.4
Systolic blood pressure (mmHg)	115.0	110.0–120.0	120.0	113.8–125.0	120.0	110.0–125.0	120.0	110.0–121.3
HDL (mg/dL)	64.2°	53.1–75.4	56.1●	49.9–65.8	64.2	55.3–71.8	63.0	54.3–72.1
Triglycerides (mg/dL)	86.2	68.4–107.3	98.3	70.6–129.1	92.1	71.3–126.7	92.1	70.9–119.1
Fasting glucose (mg/dL)	86.4	82.4–90.0	86.4	81.5–91.8	88.2	82.8–93.6	86.4	82.2–91.8

HDL: high-density lipoprotein cholesterol. Statistically significant differences (ANOVA/*t*-test; Bonferroni correction; *p* < 0.017). ° vs. CDP (commercial diet plan) group; ● vs. controls. Age: PA vs. CDP *p* < 0.001; PA vs. CG *p* = 0.003. Body mass index: PA vs. CDP—*p* < 0.001; PA vs. CG—*p* = 0.013; CG vs. CDP—*p* = 0.006. HDL: CDP vs. CG—*p* = 0.016. Physical activity: PA vs. CDP—*p* = 0.009; CDP vs. CG—*p* < 0.001. Body mass: PA vs. CDP *p* < 0.001; PA vs. CG *p* = 0.002.

**Table 2:** Correlation (r) of parameters required for calculating MetS severity.

	BMI						
Age; y	0.327	**Age**					
Systolic BP; mmHg	0.472	0.258 ^c^	**Systolic BP**				
HDL; mg/dL	−0.296	NS	NS	**HDL**			
Triglycerides; mg/dL	0.286	NS	0.171 ^b^	−0.252	**Triglycerides**		
Glucose; mg/dL	0.166 ^a^	0.227 ^d^	0.309	NS	NS	**Glucose**	
MetS%	0.706	0.258 ^e^	0.532	−0.566	0.693	0.412	**MetS%**
Z-score	0.677	0.219 ^f^	0.491	−0.527	0.656	0.385	0.960

Spearman correlation. If there is no letter following the correlation coefficient, then *p* < 0.01. The *p*-values of the numbers indicate the following: ^a^—*p* = 0.04; ^b^—*p* = 0.035; ^c^ and ^e^—*p* = 0.001; ^d^—*p* = 0.005; ^f^—*p* = 0.007. NS—not significant. Systolic BP—systolic blood pressure; BMI—body mass index; HDL—high-density lipoprotein cholesterol; Mets%—MetS percentile.

## Data Availability

Data are unavailable due to privacy and ethical restrictions.

## Abbreviations

The following abbreviations are used in this manuscript:

APOB	apolipoprotein B
APOA1	apolipoprotein A1
GL	fasting blood glucose
BMD	bone mineral density
BMI	body mass index
BP	blood pressure
CDP	commercial diet plan
CG	control group
CRP	C-reactive protein
HbA1c	glycated hemoglobin
HDL	high-density lipoprotein cholesterol
HOMA-IR	homeostatic model assessment of insulin resistance
IPAQ-SF	International Physical Activity Questionnaire Short Form
LDL	low-density lipoprotein cholesterol
MET	metabolic equivalent
MetS	metabolic syndrome
Mets%	MetS percentile
NS	not significant
PA	physical activity
SBP	systolic blood pressure
TAHSU	Tartu Applied Health Sciences University
TGs	serum triglycerides
WC	waist circumference
